# Essential Elements (Fe, Cu, Mn, Zn) in Meconium, and Newborn Length and Weight, in Relation to Maternal Lifestyle and Diet

**DOI:** 10.3390/nu17162700

**Published:** 2025-08-20

**Authors:** Bianka Mimica, Ajka Pribisalic, Zlatka Knezovic, Davorka Sutlovic

**Affiliations:** 1 Department of Gynecology and Obstetrics, University Hospital Centre Split, 21000 Split, Croatia; bmimica@kbsplit.hr; 2 Faculty of Health Sciences, University of Split, 21000 Split, Croatia; zlatka.knezovic@nzjz-split.hr (Z.K.); dsutlovic@ozs.unist.hr (D.S.); 3 Department of Public Health, School of Medicine, University of Split, 21000 Split, Croatia; 4 Teaching Institute for Public Health, Split-Dalmatia County, 21000 Split, Croatia; 5 Department of Applied Pharmacy, School of Medicine, University of Split, 21000 Split, Croatia

**Keywords:** essential elements, iron, copper, manganese, zinc, maternal diet, lifestyle, meconium

## Abstract

**Background/Objectives:** Fetal exposure to essential metals, such as iron (Fe), zinc (Zn), copper (Cu), and manganese (Mn), is influenced by maternal nutrition and lifestyle during pregnancy, potentially impacting newborn health. This study aimed to quantify concentrations of these metals in meconium and evaluate their associations, together with newborn length and weight, in relation to maternal dietary and lifestyle factors. **Methods:** This cross-sectional study included 152 mother–infant pairs recruited from various regions of Split-Dalmatia County, Croatia. Meconium samples were collected within 24 h after birth and analyzed for Fe, Zn, Cu, and Mn concentrations. Maternal characteristics, dietary intake, supplement use, and lifestyle factors were collected via structured questionnaires and supplemented by hospital records. Associations among maternal factors, meconium metal concentrations, and newborn birth weight and length were assessed using non-parametric statistical methods. **Results:** Meconium concentrations of Fe, Zn, Cu, and Mn showed substantial interindividual variability, with a strong positive correlation between Fe and Cu. Higher maternal pre-pregnancy BMI was linked to lower meconium Fe, while BMI at delivery was associated with Zn. Dietary patterns influenced metal levels: higher fruit intake was linked to increased Cu, greater vegetable intake with lower Fe, and moderate tea consumption with higher Zn. No significant associations were found with maternal smoking, residence, or supplement use. Maternal meat consumption and higher pre-pregnancy BMI were both associated with higher newborn birth weight and length. **Conclusions:** Maternal BMI and specific dietary patterns during pregnancy significantly influence essential metal concentrations in newborn meconium and are associated with newborn size, highlighting the importance of balanced maternal nutrition and healthy metabolic status during pregnancy.

## 1. Introduction

Prenatal exposure to environmental contaminants, including metals, is a growing concern due to its potential impacts on fetal development. Although many chemicals cross the placenta passively, certain metals and xenobiotics often exhibit protein-binding affinities that facilitate their active transport across the placental barrier [1]. Numerous studies have reported higher concentrations of metals in the placenta compared to maternal blood, indicating that environmental metals can penetrate the placental barrier and accumulate in the developing fetus [2,3,4].

Maternal and cord blood are often used to assess fetal exposure. However, these reflect only short-term or recent exposure and are highly influenced by the timing of sample collection. In contrast, meconium has emerged as a valuable biomarker for assessing cumulative prenatal exposure. Formed in utero over the second and third trimesters, meconium accumulates substances ingested by the fetus and provides a longer-term exposure profile, particularly to persistent substances such as trace metals [5,6,7,8,9,10].

**Figure 1:**
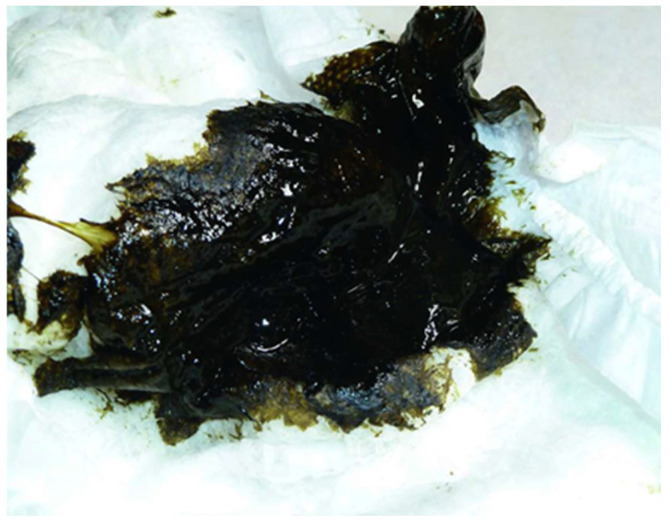
Meconium sample.

Meconium, the first stool passed by a newborn, typically within the first 24 to 48 h after birth, is composed of bile, mucus, intestinal epithelial cells, water, and materials ingested in utero, such as lanugo and amniotic fluid. It is characteristically viscous, sticky, and tar-like in texture, with a dark olive-green color and minimal odor [11] (Figure 1). Its non-invasive and easy collection, along with the provision of a sufficient sample volume for analysis and the ability to reflect long-term intrauterine exposure, makes it an ideal biomarker of fetal exposure [12].

Previous studies have successfully used meconium analysis to assess fetal metal exposure in populations residing in highly polluted environments [8,13]. Among the metals of interest, zinc (Zn), iron (Fe), copper (Cu), and manganese (Mn) are essential metals involved in critical biological processes. These include the synthesis of enzyme cofactors that support a variety of processes throughout fetal development and maintaining homeostasis throughout life [13].

Adequate Zn intake is particularly important during pregnancy, due to its involvement in cell division, protein synthesis, and overall fetal growth [14,15]. Fe is equally vital, as it supports both maternal health and normal fetal development; sufficient Fe stores are essential for proper organ formation, brain development, and optimal birth weight [16,17]. Pregnancy-related Cu is a necessary mineral for the activity of several enzymes, such as ferro-oxidases, which bind Fe to transferrin, a protein that distributes Fe. Therefore, Cu shortage might result in Fe deficiency [13,14,18]. Mn, another essential micronutrient, is involved in numerous enzymatic and metabolic functions. It is abundant in plant-based foods, and dietary intake is typically higher among vegetarians. Notably, Mn is believed to cross the placental barrier readily, making fetal exposure dependent on maternal dietary intake [13,14,19].

In addition to their biological importance, supplementation with these micronutrients during pregnancy has been associated with reduced risks of adverse outcomes, including preterm birth, low birth weight, and small-for-gestational-age (SGA) infants [17,19]. However, the extent to which fetal exposure to these elements, reflected in meconium concentrations, is associated with newborn anthropometric measures and maternal lifestyle remains underexplored.

This study aims to measure the concentrations of Zn, Fe, Cu, and Mn in meconium samples and to explore their associations, together with newborn birth weight and length, in relation to maternal lifestyle factors and dietary habits during pregnancy.

## 2. Materials and Methods

### 2.1. Respondents

The study population consisted of mother–infant pairs recruited from various regions of Split-Dalmatia County, including urban areas, the hinterland, and surrounding islands. Recruitment was conducted at the University Hospital Centre Split, Department of Gynecology and Obstetrics, between January and May 2011. During their post-delivery stay, mothers were invited to participate in the study.

A total of 275 participants were initially enrolled. However, pairs with incomplete questionnaire data or insufficient meconium samples for comprehensive analysis were excluded from the final dataset. The study was conducted prospectively, including the collection of birth size measurements, the administration of structured maternal questionnaires, and analysis of metal concentrations. To facilitate the interpretation of measured concentrations of Fe, Zn, Cu, and Mn, each mother completed a structured questionnaire. The questionnaire collected comprehensive data on maternal and infant characteristics, lifestyle factors, and dietary habits during pregnancy.

In the first half of 2025, the dataset was further enhanced with retrospective data extracted from hospital records, collected by one of the co-authors. This supplementary information included place of residence, gestational age, and number of pregnancies.

All participants provided written informed consent prior to inclusion in the study. The study received approval from the Ethics Committee of the Faculty of Medicine at the University of Split (Class: 003-08/10-03/0011; Registration number: 2181-198-04-10-0002) on 4 May 2010.

### 2.2. Samples

A total of 152 meconium samples were included for this study. Meconium samples were extracted from the infant’s diaper within the first 24 h after birth using a disposable, sterile plastic spoon and transferred into a sterile plastic container to avoid potential metal contamination. Each sample was labeled with a unique identification number matching the corresponding questionnaire and stored at −20 °C until analysis.

Prior to analysis, the samples were dried to a constant weight at 40 °C, and all results were expressed on a dry weight basis. Approximately 1 g of well-homogenized meconium sample was directly weighed into Teflon digestion tubes for the wet procedure. The digestion was performed using an automated microwave digestion system (CEM Mars 5) with a mixture of concentrated nitric acid (HNO_3_), hydrochloric acid (HCl), and hydrogen peroxide (H_2_O_2_). All reagents used were of Suprapur grade (Merck, Darmstadt, Germany).

### 2.3. Essential Metal Concentration Detection

The quantitative determination of Fe, Zn, Cu, and Mn was performed on an atomic absorption spectrometer, AAS vario6 (Analytik Jena, Germany), equipped with deuterium background correction. Measurements were performed in flame mode using an acetylene/air mixture. The limits of detection (LOD), calculated from standard deviations of the blank measurements, were 0.01 mg/L for Zn and Mn, and 0.05 mg/L for Fe and Cu. Each sample was measured five times, and the coefficients of variation for all metals were less than 5%.

Method accuracy was verified using the certified reference material Seronorm Trace Elements Urine L-2 (lot no. 210705, Sero AS, Hvalstad, Norway). The obtained values showed good agreement with the certified values, more precisely, 101.6% for Zn. For the other elements, accuracy was evaluated using a spiking method with standard solutions, yielding compliances of 96.8% for Fe, 102.4 for Cu, and 98.4% for Mn.

Additionally, accuracy during measurements was routinely checked using standard solutions of known concentrations analyzed as samples. Standard solutions of Zn, Fe, Cu, and Mn for measurements were prepared from Merck (Darmstadt, Germany) stock solutions (1000 ± 2 mg/L, Suprapur).

### 2.4. Maternal Lifestyle and Diet

Information on maternal lifestyle and anthropometric characteristics was collected through a structured questionnaire, supplemented by data extracted from medical records. Variables assessed included maternal age (in years), place of residence (categorized as rural area, small town, industrial town, or larger city), and smoking status (recorded as non-smoker, up to 10 cigarettes per day, or 10 to 20 cigarettes per day). The use of dietary supplements during pregnancy was also recorded (yes/no).

Anthropometric variables included maternal length, pre-pregnancy weight, and weight at delivery. Based on these measurements, pre-pregnancy BMI, BMI at delivery, and total gestational weight gain were calculated as continuous variables. BMI was further classified into four categories: underweight (<18.5 kg/m^2^), normal weight (18.5–24.9 kg/m^2^), overweight (25.0–29.9 kg/m^2^), and obese (≥30.0 kg/m^2^).

Gestational age at delivery (in weeks) was documented for each participant. In addition, newborn anthropometric measurements, including birth length (in centimeters) and birth weight (in grams), were obtained from medical records for subsequent analysis.

Maternal dietary intake during pregnancy was assessed using a structured questionnaire that included questions on the weekly consumption frequency for a range of food groups and beverages. Food categories included fish, meat, fruit, vegetables, cereals, and milk, while beverage items comprised tea, coffee, wine, and hard alcoholic beverages. For each category, mothers indicated their consumption as “never,” “occasionally,” or “frequently/every day.” All responses were categorized accordingly for subsequent analysis.

### 2.5. Statistical Analysis

The normality of the data was assessed using the Shapiro–Wilk test, which was chosen due to the sample size (N = 152) and its greater sensitivity for smaller samples (N < 200). Descriptive statistics were reported as means with standard deviations (SDs) or medians with interquartile ranges (IQRs), as appropriate. Spearman’s rank correlation coefficient was employed to assess the strength and direction of associations between the measured concentrations of essential metals.

Boxplots were generated to visualize the distributions, providing information on median values, interindividual variability, and the presence of potential outliers. Group comparisons for continuous variables were performed using the Mann–Whitney U test when comparing two independent groups, and the Kruskal–Wallis test was used for three or more independent groups. When statistically significant differences were identified, the Mann–Whitney U test was subsequently utilized as a post hoc analysis to determine specific between-group differences.

All statistical analyses were performed using IBM SPSS version 21. Statistical significance was set at *p* < 0.050.

## 3. Results and Discussion

### 3.1. Characteristics of the Study Sample

The following variables were normally distributed: mother’s age, BMI at delivery, weight gain, and newborn’s birth weight. Variables that deviated from normality were pre-pregnancy BMI, gestational age, and the newborn’s birth length.

The mean age of mothers was 30.12 years (SD = 0.43). The mean BMI at delivery was 27.80 (SD = 0.28), and the average weight gain during pregnancy was 15.68 kg (SD = 0.38). The mean birth weight of newborns was 3 576.09 g (SD = 37.51). Table 1 presents the baseline characteristics of the study population, with all continuous variables reported as medians and IQRs, regardless of their distribution.

Most women in this study were first-time mothers (50%, had one child), while about a third (33.6%, n = 51) had two children. A smaller number had three children (10.5%), and just a handful had four or more, with up to as many as nine children. Regarding pregnancy history, nearly half of the women (47%, n = 72) had been pregnant once, about 29% (n = 44) twice, and 13% (n = 20) three times. A small number had gone through four or more pregnancies, with one participant reporting as many as ten.

### 3.2. Maternal Diet Characteristics

**Figure 2:**
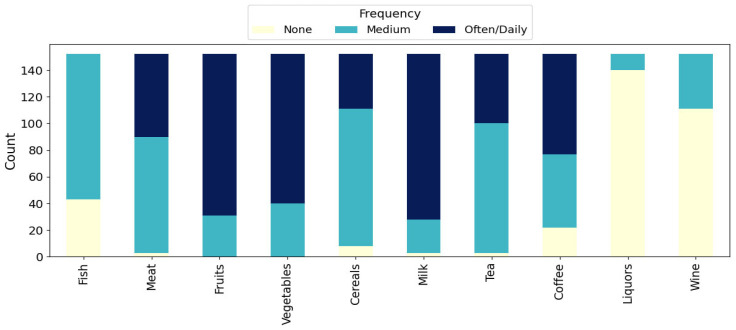
Weekly consumption frequencies of food and beverage categories (N = 152).

The stacked bar chart in Figure 2 shows the weekly consumption frequencies for various food and beverage categories among the mothers. Among the 152 mothers, frequent (often/daily) intake was most prevalent for milk (n = 124, 81.6%), fruits (n = 121, 79.6%), and vegetables (n = 112, 73.7%), with no mother reporting no consumption in these categories. Cereals, meat, and fish were mainly consumed at moderate frequencies, with 111 mothers (73.0%) reporting moderate cereal intake, 87 (57.2%) reporting moderate meat intake, and 109 (71.7%) moderate fish intake. Tea was also consumed in moderate frequencies (n = 97, 63.8%). For coffee, moderate (55, 36.2%) and frequent (75, 49.3%) consumption were both common. In contrast, hard alcoholic beverages (liquors) and wine were rarely consumed, as 140 mothers (92.1%) reported no consumption of liquors and 111 (73.0%) reported no wine consumption during the week.

These results highlight a pattern of habitual consumption of plant-based foods and dairy, moderate intake of meat, fish, and cereals, and generally low use of alcoholic beverages within the study group.

### 3.3. Meconium Metal Concentrations

**Figure 3:**
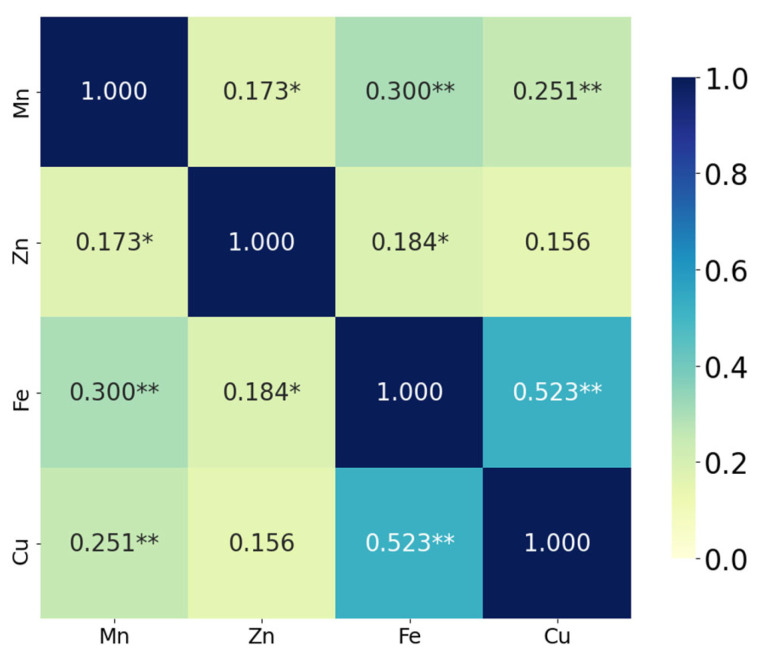
Spearman correlation heatmap of meconium metal concentrations (Mn, Zn, Fe, and Cu). Significance level is denoted with * (* *p* < 0.05, ** *p* < 0.01).

The correlation analysis demonstrated that Fe and Cu exhibited the strongest positive correlation (ρ = 0.523, *p* < 0.001), suggesting a significant co-variation between these two elements. Mn was also moderately and significantly correlated with both Fe (ρ = 0.300, *p* < 0.001) and with Cu (ρ = 0.251, *p* = 0.002). In addition, a weaker but statistically significant association was observed between Mn and Zn (ρ = 0.173, *p* = 0.033), as well as between Zn and Fe (ρ = 0.184, *p* = 0.023). The correlation between Zn and Cu (ρ = 0.156) did not reach statistical significance (*p* = 0.055) (Figure 3).

These findings suggest that some micronutrients, particularly Fe and Cu, vary together in a biologically or environmentally meaningful way, which may reflect shared uptake mechanisms, similar sources, or co-regulation processes [17,18].

To provide further context, the distribution of individual meconium metal concentrations was examined. As presented in Table 2, Mn, Zn, Fe, and Cu levels displayed substantial variability among participants, exhibiting broad ranges, distinct median values, and differing interquartile intervals.

### 3.4. Meconium Metal Concentrations in Relation to Maternal Lifestyle Factors

#### 3.4.1. Meconium Metal Concentrations in Relation to Maternal Supplement Use During Pregnancy

**Figure 4:**
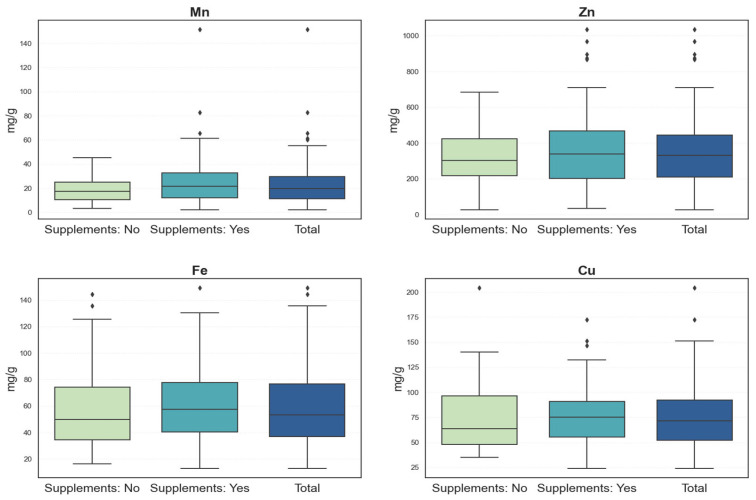
Boxplots of Mn, Zn, Fe, and Cu concentrations by maternal supplement use status (N = 152).

Figure 4 presents box plots of each metal concentration for the total sample, as well as stratified by supplement use. Considerable variability and frequent outliers were observed across both groups, reflecting heterogeneous exposure or accumulation levels.

Among participants who did not use dietary supplements, the medians (IQRs) were 17.18 mg/g (16.01) for Mn, 301.71 mg/g (212.33) for Zn, 49.98 mg/g (40.44) for Fe, and 63.65 mg/g (48.54) for Cu. Supplement users showed slightly higher concentrations, with median (IQR) values of 21.35 mg/g (21.14) for Mn, 339.03 mg/g (285.70) for Zn, 57.40 mg/g (38.85) for Fe, and 75.35 mg/g (37.63) for Cu.

While supplement users generally exhibited higher concentrations, all distributions showed wide variability and substantial overlap between groups, as illustrated in Figure 4. Mean rank values also supported this pattern, with slightly higher ranks observed for supplement users across all metals. However, none of these differences reached statistical significance at the *p* < 0.05 level (Mn: U = 2196.50, *p* = 0.060; Zn: U = 2494.00, *p* = 0.459; Fe: U = 2421.00, *p* = 0.308; Cu: U = 2381.50, *p* = 0.242). Therefore, while supplement users tended to have higher median metal concentrations, these differences were not statistically significant and should be interpreted with caution to avoid overestimating any potential association. This lack of statistical difference may, in part, reflect the substantial interindividual variability in metal concentrations observed in meconium. Such variability has been well-documented in previous research and is often attributed to a combination of exposure sources, nutrient bioavailability, genetic factors, and environmental influences [13].

Given this complexity, the influence of dietary supplementation on elemental concentrations in meconium remains nuanced. Some authors report that supplementation during pregnancy can increase concentrations of certain micronutrients, such as Fe, Zn, and Mn, in biological samples of both mothers and newborns, but may also affect homeostatic relationships with other elements, such as Cu [20]. In addition, supplementation has been shown to mitigate the adverse effects of toxic metal exposure, such as cadmium and lead, further supporting the importance of targeted nutritional interventions during pregnancy [21].

However, the presence of outliers, even among mothers who did not use supplements, suggests the involvement of other exposure pathways, such as diet, drinking water, environmental pollutants, or individual differences in nutrient absorption, also reported in the literature [22]. These findings highlight the importance of continued research on the interactions among supplementation, environmental factors, and the accumulation of elements in meconium.

#### 3.4.2. Meconium Metal Concentrations in Relation to Maternal Residential Locations During Pregnancy

Mean ranks for Mn, Zn, Fe, and Cu concentrations showed only minor variation between groups, and none of the group differences were statistically significant (Mn: H(3) = 1.44, *p* = 0.697; Zn: H(3) = 1.53, *p* = 0.674; Fe: H(3) = 0.92, *p* = 0.820; Cu: H(3) = 1.51, *p* = 0.680). These results indicate that metal concentrations did not significantly differ by place of residence during pregnancy.

These results align with previous studies indicating that, in populations not exposed to notable industrial pollution, differences in trace metal concentrations between rural and urban environments tend to be minimal or insignificant. For example, a study conducted in the United States reported slightly elevated Cu levels among urban-dwelling pregnant women, whereas Mn, Zn, and Fe concentrations showed no variation across residential environments [23].

In contrast, studies in industrially affected regions, such as communities located near refineries, mining sites, or chemical processing facilities, have consistently demonstrated elevated levels of both toxic and essential metals in biological samples, including meconium. In such a context, increased metal concentrations are frequently associated with residential proximity to pollution sources and parental occupational exposure [24].

#### 3.4.3. Meconium Metal Concentrations in Relation to Maternal Smoking During Pregnancy

For Mn, mean ranks decreased with higher smoking, while, for the other metals, the pattern varied (e.g., for Zn, the mean rank was highest in the heaviest smoking group). However, none of the differences reached statistical significance (Mn: H(2) = 0.92, *p* = 0.631; Zn: H(2) = 1.19, *p* = 0.552; Fe: H(2) = 2.83, *p* = 0.242; Cu: H(2) = 5.25, *p* = 0.072). Thus, no statistically significant differences in metal concentrations by maternal smoking status were identified.

Although some variation in metal concentrations was observed among mothers from different smoking categories, the data do not indicate a clear association between the number of cigarettes smoked during pregnancy and metal levels in newborn’s meconium. The absence of statistically significant differences may reflect limitations such as the relatively small sample size, substantial interindividual variability in metal metabolism, or the influence of confounding factors affecting fetal metal accumulation. It is also possible that more pronounced differences would emerge specifically for toxic metals known to be directly associated with smoking. Moreover, the effects of smoking on specific metal concentrations may be element-specific and biologically complex, with opposing mechanisms influencing different metals, further obscuring consistent patterns.

These findings are consistent with previous studies indicating that, while maternal smoking can increase exposure to toxic metals such as cadmium and lead, its effect on essential metal concentrations in meconium is often inconsistent or statistically insignificant [25]. Conversely, some studies suggest decreased levels of magnesium and Zn levels in samples from newborns whose mothers smoked during pregnancy, suggesting potential implications for fetal development [26].

#### 3.4.4. Meconium Metal Concentrations in Relation to Pre-Pregnancy BMI Category

The mean ranks for Mn, Zn, Fe, and Cu varied among BMI categories, with the highest mean ranks for Mn and Fe in the normal-weight and overweight groups, respectively. The test indicated a statistically significant difference in Fe concentrations between BMI groups (H(3) = 8.89, *p* = 0.031). However, differences were not significant for Mn (H(3) = 3.39, *p* = 0.336), Zn (H(3) = 1.48, *p* = 0.688), or Cu (H(3) = 2.46, *p* = 0.482). These findings suggest that pre-pregnancy BMI category may be associated with differences in iron concentrations, but not in those of Mn, Zn, or Cu.

Newborns of obese mothers had significantly lower Fe levels compared to those of normal-weight mothers (U = 183.0, *p* = 0.014), overweight mothers (U = 17.0, *p* = 0.007), and underweight mothers (U = 12.0, *p* = 0.016). No statistically significant differences were found in other pairwise comparisons involving Fe (all *p* > 0.050).

These findings suggest that pre-pregnancy BMI may influence fetal iron accumulation or its metabolism, potentially mediated by various factors, such as maternal nutritional status, inflammatory processes, or altered placental transport of elements associated with different BMI categories.

Similar associations between maternal BMI and fetal iron status have been reported in prior studies. Specifically, overweight and obesity have been linked to disruptions in iron metabolism, including an increased risk of fetal anemia or iron overload, which may result from inflammatory responses or placental transport dysfunction [27,28].

#### 3.4.5. Meconium Metal Concentrations in Relation to BMI Category at Delivery

The mean ranks for Mn, Zn, Fe, and Cu concentrations in meconium varied across BMI categories at delivery. The highest mean ranks for Fe and Cu were observed in the normal-weight group, while the highest ranks for Mn and Zn were found in the overweight group. The test revealed a statistically significant difference in Zn concentrations between BMI groups (H(2) = 7.03, *p* = 0.030), while differences in Fe concentrations approached statistical significance (H(2) = 5.44, *p* = 0.066). However, no significant differences were observed for Mn (H(2) = 2.17, *p* = 0.339) or Cu (H(2) = 2.49, *p* = 0.289).

A statistically significant difference was observed between the normal- and overweight groups (U = 876.00, *p* = 0.030), with higher Zn levels in the overweight group compared to the normal-weight group. Additionally, a significant difference was found between the overweight and obese groups (U = 1290.00, *p* = 0.036), again with higher Zn levels in the overweight group. No significant difference in Zn concentrations was observed between the normal-weight and obese groups (*p* = 0.749).

### 3.5. Meconium Metal Concentration in Relation to Maternal Diet

For fruit intake, a significant group difference was observed for Cu concentrations (H(1) = 6.03, *p* = 0.014), with higher Cu levels among those reporting more frequent fruit consumption compared to those who did not consume fruit. Furthermore, mothers who reported moderate fruit consumption had a higher mean rank of Cu than those who reported often/daily consumption, indicating that Cu levels were higher among mothers with moderate fruit intake compared to those with daily intake. Group differences for Mn, Zn, and Fe in relation to fruit intake did not reach statistical significance (all *p* > 0.050).

Vegetable consumption was significantly associated with Fe concentrations (H(1) = 6.29, *p* = 0.012), with higher vegetable intake corresponding to lower median Fe levels. Group differences for Mn, Zn, and Cu in relation to vegetable intake did not reach statistical significance (all *p* > 0.050).

Zinc was significantly associated with tea consumption (H(2) = 9.78, *p* = 0.008), indicating variability in Zn levels depending on tea intake frequency. Specifically, participants who reported moderate tea consumption had the highest mean rank for zinc, followed by those with high intake and those with no intake. Further pairwise comparisons showed that mothers who consumed tea at a moderate frequency had significantly higher Zn concentrations than both those who reported no tea consumption (*p* = 0.036) and those with daily or frequent tea intake (*p* = 0.014). Other metals did not differ by tea consumption (all *p* > 0.050).

No significant differences in meconium metal concentrations were found according to consumption of fish, meat, cereals, milk, coffee, wine, or hard alcoholic beverages (all *p* > 0.050).

Specifically, higher fruit intake was associated with significantly higher Cu levels, while greater vegetable consumption was associated with increased Fe concentrations. Tea intake demonstrated a more complex relationship with Zn, with the highest Zn concentrations observed among participants with moderate tea consumption.

These findings are consistent with the existing literature, supporting the influence of dietary patterns on essential metal status during pregnancy, potentially reflecting changes in nutrient intake or bioavailability. For example, fruits and vegetables are established dietary sources of Cu and Fe, and their increased consumption may enhance the availability of these elements to the fetus [29]. Tea, meanwhile, is a recognized source of essential trace elements, such as Zn, Cu, and Fe, important for various physiological functions and fetal development [30]. Our observation of higher meconium zinc levels with maternal tea consumption is supported by studies showing that red and yellow teas, especially those from China, contain the highest Zn concentrations compared to other varieties [31]. Although we did not gather data on specific tea types, these differences suggest that tea variety may influence Zn intake. Tea also contains polyphenols and tannins that can affect the absorption of zinc and other minerals; however, this effect may be nonlinear and depend on consumption frequency, as seen in our sample [32]. Furthermore, maternal and cord blood zinc levels have been positively correlated with birth weight, highlighting Zn’s critical role during pregnancy [33]. Together, these findings underscore the potential benefits of moderate tea consumption for maternal and neonatal Zn status.

### 3.6. Newborn Length and Weight in Relation to Maternal Diet

Statistically significant differences were observed in both newborn weight and newborn length for meat consumption during pregnancy (weight: H(2) = 6.11, *p* = 0.047; length: H(2) = 6.29, *p* = 0.043).

Newborns of mothers who reported no meat intake during pregnancy had significantly lower birth weights (U = 20.0, *p* = 0.022) and lengths (U = 21.0, *p* = 0.021) compared with those whose mothers reported frequent meat consumption. Among mothers who consumed no meat, compared to those with moderate intake, birth weight and length were again lower in the no-meat group. The difference approached significance for birth weight (U = 44.5, *p* = 0.053), and was significant for birth length (U = 33.5, *p* = 0.026). No significant differences in birth weight or length were observed between the moderate and frequent meat consumption groups (all *p* > 0.050).

No statistically significant differences in newborn weight or length were found in relation to fish, fruit, vegetable, cereal, milk, tea, coffee, wine, or hard alcoholic beverage consumption during pregnancy (all *p* > 0.050).

While statistically significant associations were observed between maternal meat intake during pregnancy and newborn weight and length, it is important to interpret these findings cautiously. Across pairwise comparisons, abstaining from meat during pregnancy was consistently associated with lower newborn birth weight and length, with the largest differences observed between non-consumers and frequent meat consumers.

That associations may be attributed to the fact that meat is a rich source of high-quality protein, iron, and other essential nutrients critical for fetal growth and development [34]. Inadequate meat consumption could result in reduced intake of these key nutrients, potentially impairing fetal development and ultimately the newborn’s size. Conversely, the absence of significant differences in birth weight and length related to the intake of other food groups may reflect the complex influence of diet during pregnancy, wherein specific nutrients predominantly found in meat may play a dominant role in fetal growth [35].

However, the observed trend toward higher birth weight and length among infants born to mothers with higher BMI aligns with previous findings that maternal overweight and obesity are often associated with increased newborn size and birth weight [36,37]. The lack of a significant difference in this sample may be due to the sample size or variability within the groups.

Nevertheless, meat intake may serve as a proxy for multiple nutritional and socioeconomic factors that were not fully accounted for in this study. The observed associations may reflect differences in overall diet quality, nutrient availability, or lifestyle variables linked to meat consumption. Further research with more detailed dietary assessments, including types of meat, and consideration of additional confounding factors, is needed to better understand the specific role of meat intake in fetal growth and anthropometric outcomes.

### 3.7. Newborn Length and Weight in Relation to Maternal Lifestyle

There were no statistically significant differences in newborn weight or length based on the mother’s place of residence during pregnancy (H(3) = 3.68, *p* = 0.298 for weight; H(3) = 1.68, *p* = 0.640 for length), smoking status during pregnancy (H(2) = 1.12, *p* = 0.573 for weight; H(2) = 0.33, *p* = 0.848 for length), BMI category at delivery (H(2) = 3.80, *p* = 0.150 for weight; H(2) = 1.38, *p* = 0.503 for length), or the use of dietary supplements (H(1) = 0.04, *p* = 0.846 for weight; H(1) = 0.01, *p* = 0.977 for length).

However, a significant difference in newborn length was observed across categories of pre-pregnancy BMI (H(3) = 8.41, *p* = 0.038), while the difference in newborn weight approached significance (H(3) = 7.13, *p* = 0.068). Children of mothers with higher pre-pregnancy BMI tended to have higher mean ranks for both weight and length, indicating a potential association between maternal pre-pregnancy BMI and newborn anthropometric outcomes.

Pairwise comparisons identified significant differences between underweight and normal-weight mothers, where newborns of normal-weight mothers had significantly higher weight (U = 329.00, *p* = 0.007) and length (U = 327.50, *p* = 0.006). Additionally, newborns of mothers in the overweight category were significantly longer than those of underweight mothers (U = 39.00, *p* = 0.009), and the difference in weight approached significance (U = 54.50, *p* = 0.066). A difference in newborn length between normal-weight and obese mothers was significant (U = 17.00, *p* = 0.040), with a borderline statistically significant difference in weight (U = 18.00, *p* = 0.063), with newborns of obese mothers having higher values. No statistically significant differences in either weight or length were observed when comparing other BMI category pairs (all *p* > 0.050).

The results indicate that lifestyle factors, such as the maternal place of residence, smoking status during pregnancy, and dietary supplement use, did not significantly influence newborn weight and length in this sample. This finding is consistent with previous studies reporting inconsistent or weak associations between these factors and newborn anthropometric outcomes [38,39].

However, significant differences in newborn length and nearly significant differences in weight were observed across maternal pre-pregnancy BMI categories. Newborns of mothers with higher pre-pregnancy BMI values tended to have greater size measurements, supporting earlier findings that maternal overweight and obesity are associated with birth weight and length [40]. Moreover, significant pairwise differences between BMI categories suggest that varying degrees of overweight and obesity may influence fetal growth through different mechanisms, such as hormonal alterations and inflammatory pathways associated with excess maternal adiposity [41].

### 3.8. Study Limitations

This study has several limitations that should be considered when interpreting its findings. First, the sample size, while sufficient for overall analyses, was modest for some subgroup comparisons and may have limited the statistical power to detect smaller differences. The observational, cross-sectional design precludes definitive conclusions about causality among maternal exposures, dietary habits, and measured outcomes. Data on maternal diet, supplement use, and lifestyle factors were based on self-reported questionnaires, which are subject to recall bias and possible misclassification. The dietary questionnaire used in this study was structured, but not formally validated, which may affect the accuracy of reported dietary intakes and the interpretation of associations; future research should consider using validated tools or conducting validation to enhance data reliability. Additionally, multiple statistical comparisons were performed without formal correction for multiple testing, such as the Bonferroni adjustment. This increases the risk of type I errors, and some statistically significant findings may be due to chance. Therefore, the results should be interpreted with caution. Furthermore, this study did not include adjusting for potential confounders, which may have influenced the observed associations. Therefore, future research with larger sample sizes, confounder adjustments, and appropriate multiple testing corrections is needed to confirm these associations and enhance the robustness of conclusions.

Because all participants were recruited from a single regional hospital in Split-Dalmatia County, the generalizability of the results to broader or more diverse populations may be limited. This study also did not account for all possible environmental or occupational exposures to metals, or unmeasured confounding, such as genetic factors or detailed placental function, which may have influenced results. Finally, only selected essential metals (Mn, Zn, Fe, and Cu) were analyzed, so the influences of other elements or contaminants on fetal development could not be assessed. These limitations highlight the need for further research using larger, more diverse cohorts and comprehensive, prospective data collection to better understand the determinants of fetal metal exposure and newborn outcomes.

## 4. Conclusions

This study comprehensively examined the relationships among maternal lifestyle factors, dietary habits, and essential metal concentrations in newborn meconium, alongside newborn anthropometric outcomes. The findings demonstrate considerable interindividual variability in meconium concentrations of Fe, Zn, Cu, and Mn, with significant positive correlations, notably between Fe and Cu, suggesting shared regulatory or uptake mechanisms.

Maternal dietary patterns emerged as influential determinants of elemental status; higher fruit intake was associated with increased Cu levels, greater vegetable consumption corresponded with lower Fe concentrations, and moderate tea intake was linked to elevated Zn levels. Among lifestyle factors, maternal pre-pregnancy BMI exhibited a significant association with fetal iron accumulation, with newborns of obese mothers displaying lower Fe concentrations. Additionally, maternal BMI category at delivery was related to variations in Zn levels. In contrast, maternal smoking, place of residence, and dietary supplement use did not significantly influence metal concentrations in meconium.

Regarding newborn anthropometric measurements, only meat consumption during pregnancy showed a clear positive association with birth weight and length, underscoring the critical role of high-quality protein and micronutrient intake in fetal growth. Furthermore, maternal pre-pregnancy BMI was significantly related to newborn size, with infants born to mothers of higher BMI categories tending toward greater birth weight and length, consistent with the existing literature linking maternal adiposity to enhanced fetal growth.

Overall, these findings highlight the complex interplay among maternal nutrition, metabolic status, and fetal exposure to essential trace elements. They emphasize the importance of maintaining adequate maternal nutritional status, including balanced dietary intake and healthy BMI, for optimizing fetal growth and micronutrient transfer. Further research is warranted to elucidate the mechanistic pathways underlying these associations and to explore potential interventions aimed at improving maternal–fetal micronutrient status and birth outcomes.

## Figures and Tables

**Table 1:** Maternal and newborn characteristics, N = 152.

Age (years); median (IQR)	31.00 (8.00)
Place of residence; n (%)	
Rural area	42 (27.60)
Small town	29 (19.10)
Industrial town	19 (12.50)
Larger city	62 (40.80)
Smoking status; n (%)	
Non-smoker	115 (75.70)
Up to 10 cigarettes per day	28 (18.40)
10 to 20 cigarettes per day	9 (5.90)
Consumption of dietary supplements (yes); n (%)	96 (63.2)
BMI pre-pregnancy; median (IQR)	22.10 (3.69)
BMI at delivery; median (IQR)	27.62 (4.54)
Weight gain (kg); median (IQR)	15.00 (7.00)
Gestation (weeks); median (IQR)	40 (1)
Newborn length(cm); median (IQR)	51.00 (2.00)
Newborn weight (g); median (IQR)	3 580 (635)

BMI—body mass index; IQR—interquartile range.

**Table 2:** Summary of metal concentration levels (mg/g) in meconium samples.

Metal	Minimum (mg/g)	Maximum (mg/g)	Median (mg/g)	IQR (mg/g)
Manganese (Mn)	1.93	151.60	19.44	19.14
Zinc (Zn)	25.30	1035.22	331.40	235.69
Iron (Fe)	12.81	149.40	53.45	40.63
Copper (Cu)	24.06	204.40	71.67	40.35

IQR—interquartile range.

## Data Availability

The data presented in this study are available upon request from the corresponding author.
